# *SF3B1* mutations in AML are strongly associated with *MECOM* rearrangements and may be indicative of an MDS pre-phase

**DOI:** 10.1038/s41375-022-01734-7

**Published:** 2022-10-21

**Authors:** Sandra Huber, Torsten Haferlach, Manja Meggendorfer, Stephan Hutter, Gregor Hoermann, Constance Baer, Wolfgang Kern, Claudia Haferlach

**Affiliations:** MLL Munich Leukemia Laboratory, Max-Lebsche-Platz 31, 81377 Munich, Germany

**Keywords:** Cancer genomics, Acute myeloid leukaemia

## To the Editor:

In AML *SF3B1* mutations are recurrently found, most frequently in AML-MRC [1] and were shown to be highly specific for secondary AML (s-AML) arising post MDS or MDS/MPN [2]. Thus, the presence of *SF3B1* mutations is considered as diagnostic criteria for AML-MR according to the 5th edition of the WHO classification (WHO 2022; [3]). Here, we address the prognostic impact of *SF3B1* mutations in AML and evaluate the genetic landscape of *SF3B1* mutated patients at AML diagnosis and during follow-up.

Based on the revised 4th edition of the WHO classification (WHO 2017), AML are classified into AML with recurrent genetic abnormalities, AML with myelodysplasia-related changes (AML-MRC) or AML, not otherwise specified (AML-NOS) [4]. Several changes are announced in the WHO 2022 [3] incorporating more genetically defined entity criteria. For example, AML with mutated *RUNX1* is no longer recognized as distinct entity, AML-MRC is replaced by AML-MR considering gene mutations while removing morphologic criteria and AML sub-groups with rearranged *KMT2A* or *MECOM* are extended including all partner genes.

Within the last years, many prognostically relevant driver genes in AML have been identified including also spliceosome genes [5]. In myeloid malignancies, *SF3B1* is most frequently mutated in MDS or MDS/MPN and associated with a favorable prognosis and an indolent disease course [6–8]. More recent data by Bernard et al. indicate that the favorable outcome is restricted to those patients lacking co-mutations in *BCOR*, *BCORL1*, *NRAS*, *RUNX1*, *SRSF2*, *STAG2* and del(5q) [9].

For this analysis we selected 735 AML samples with material available to perform whole genome sequencing sent to the MLL Munich Leukemia Laboratory between 09/2005 and 01/2020. Therapy-related AML were excluded from this study. Within the cohort 89% (652/735) were de novo AML cases and 11% (83/735) s-AMLs. For further details on cohort and statistics see Supplementary Methods. All cases were classified into specific sub-groups according to the currently used WHO 2017 [4]. *SF3B1*^mut^ cases were further classified according to WHO 2022 [3] and the International Consensus Classification (ICC; [10]). For abbreviations of entities, see Supplementary Table S1. All patients gave their written informed consent for genetic analyses and to the use of laboratory results and clinical data for research purposes according to the Declaration of Helsinki. The study was further approved by the laboratory´s institutional review board. All samples were subjected to whole genome and targeted panel sequencing (Supplementary Methods).

**Fig. 1:**
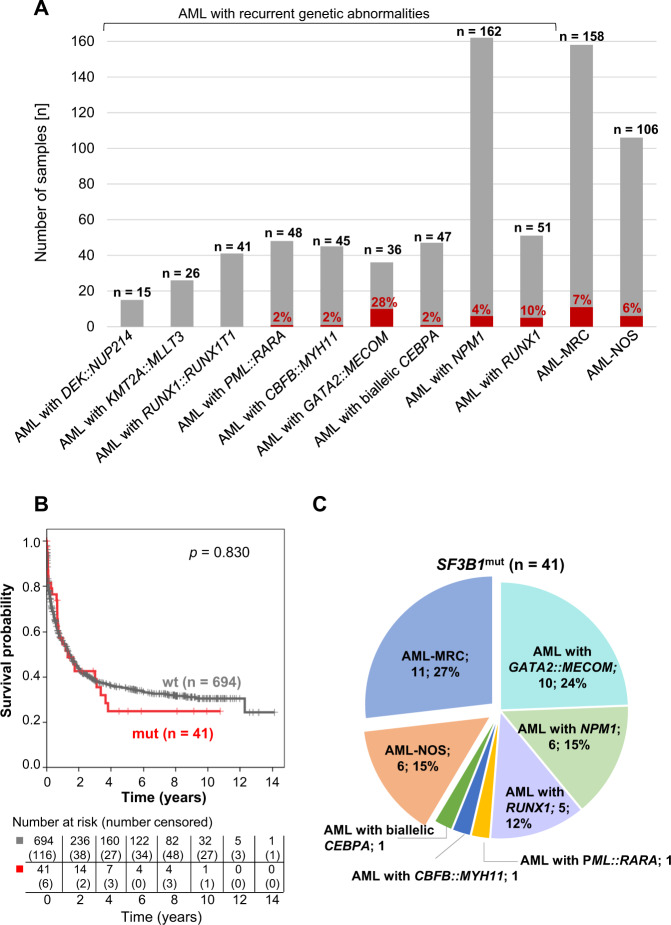
Distribution and OS of *SF3B1* mutations in AML. **A** *SF3B1* mutation status within different AML entities (red: mutated; gray: wild-type). **B** OS of patients with mutated (*n* = 41; red) vs. wild-type (*n* = 694; gray) *SF3B1* within the entire AML cohort. **C** WHO 2017 entities of *SF3B1* mutated AML (*n* = 41).

We identified *SF3B1* mutations in a small fraction (6%; 41/735) of AML patients (Fig. 1A and Supplementary Table S1) in line with published results [5, 11]. Based on WHO 2017, *SF3B1* mutations were found in AML with recurrent genetic abnormalities (24/471; 5%), AML-MRC (11/158; 7%) and AML-NOS (6/106; 6%) (Fig. 1A). Within the entire AML cohort, comprising samples from 16 different entities, *SF3B1* mutations were detected in eight different AML entities (Supplementary Table S1), most frequently within AML with *GATA2::MECOM* (10/36; 28%), thereby confirming the association of *SF3B1* mutations with *GATA2::MECOM* rearrangements as previously published [12]. Notably, within AML-NOS *SF3B1* mutations were exclusively found in samples diagnosed with AML with maturation (Supplementary Table S1). The presence of ring sideroblasts in *SF3B1*^mut^ AML is described in the Supplementary Results. *SF3B1* mutations did not affect OS in the total AML cohort (median: 16 vs. 17 months; *p* = 0.830; Fig. 1B). Within all 41 *SF3B1*^mut^ cases AML-MRC (11/41; 27%) and AML with *GATA2::MECOM* (10/41; 24%) were most frequent (Fig. 1C). When stratified for AML sub-entities, there was also no impact of *SF3B1* mutations on OS within each sub-entity (Supplementary Fig. S1A–E), however OS was different within *SF3B1*^mut^ AML if stratified according to WHO entities (Supplementary Fig. S1F, G). Thus, the prognosis of the *SF3B1*^mut^ AML seems to be dominated by the sub-entity, concordant with a previous report showing that splicing mutations (including *SF3B1*) per se are not prognostic in AML [13].

**Fig. 2:**
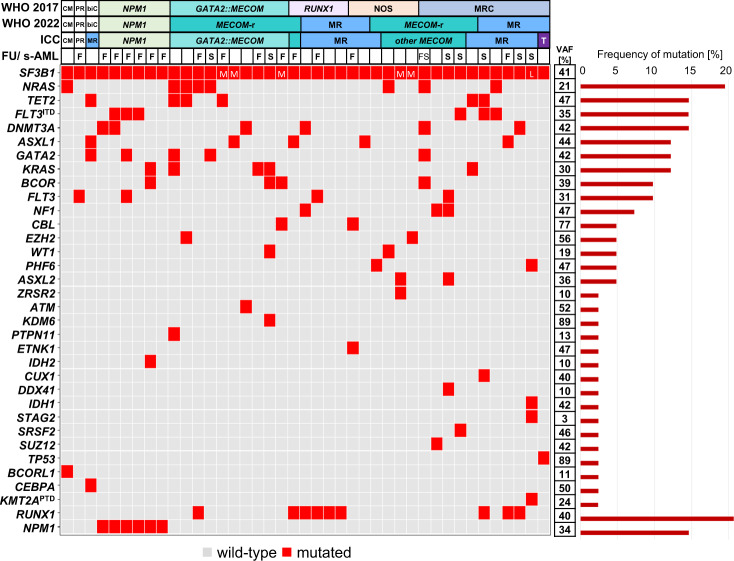
Molecular characterization of AML patients with mutated *SF3B1*. Illustration of all 41 samples, each column represents one patient. Genes (gray: wild-type; red: mutated) as well as the WHO and ICC entities are given for each patient. Secondary AMLs (s-AMLs) are marked with “S” and those with available follow-up (FU) data with “F”. VAF variant allelic frequency (mean), CM *CBFB::MYH11*, PR *PML::RARA*, biC biallelic *CEBPA*, NOS not otherwise specified, MR(C) myelodysplasia-related (changes), *MECOM*-r *MECOM* rearrangement, T mutated *TP53*, L low VAF (0–14%), M medium VAF (15–29%); Remaining cases showed *SF3B1* VAFs ≥30%.

In the total cohort, *SF3B1* mutations showed a mean variant allelic frequency (VAF) of 41% and those mutations affecting amino acids K666 and K700 were found most frequently (Supplementary Results and Supplementary Fig. S2) similar to previous studies [13, 14]. On average, *SF3B1*^mut^ patients harbored 3.3 mutations (AML-NOS: 2.5; AML with *RUNX1*: 2.8; AML with *GATA2::MECOM*: 3.3; AML-MRC: 3.6; AML with *NPM1*: 3.7; Fig. 2). The most frequent additional mutations in *SF3B1*^mut^ patients were *RUNX1* (9/41; 22%) and *NRAS* (8/41; 20%). *NPM1, TET2*, or *DNMT3A* mutations or *FLT3*-ITD were detected in 15% (6/41) each. *RUNX1* mutations were present besides within AML with *RUNX1* mutation, also in AML-MRC (*n* = 3) and AML with *GATA2::MECOM* (*n* = 1). Interestingly, 37% (15/41) of *SF3B1*^mut^ patients harbored at least one mutation in a DTA gene (*DNMT3A, TET2, ASXL1*). Additional mutations were found in 5 to 21 different genes depending on the respective entity (Supplementary Fig. S3A–E). Within *SF3B1*^mut^ patients 10 cases showed *MECOM* rearrangements (*MECOM*-r) with a different partner gene than *GATA2*. This resulted in 49% (20/41) of *SF3B1*^mut^ patients harboring a *MECOM*-r (Fig. 2). Conversely, 31% (20/64) of all AML with *MECOM*-r showed an *SF3B1* mutation, which was thus the second most frequent mutation within this AML entity after *NRAS* mutations (36%; 23/64). *SF3B1* mutations were significantly associated with *MECOM*-r (31% [20/64] vs. 3% [21/671]; *p* < 0.001). In summary, the majority (78%, 32/41) of *SF3B1*^mut^ AML were either AML with *MECOM*-r (*n* = 20) or AML-MR (*n* = 12), underpinning the strong association of *SF3B1* mutations with these two entities (Fig. 2; further details on the classification of *SF3B1*^mut^ cases are provided in the Supplementary Results). A prior history of MDS or MDS/MPN was documented in 20% (8/41) of *SF3B1*^mut^ patients harboring on average 4.3 mutations at AML diagnosis (Fig. 2 and Supplementary Fig. S4A). Thereof, 63% (5/8) had a *MECOM*-r and 25% showed *RUNX1, DNMT3A, GATA2, NRAS, BCOR* mutations or *FLT3*-ITD when AML was diagnosed. The *SF3B1*^mut^ was already present in the prior MDS stage in 4/5 patients with available MDS data (Supplementary Results and Supplementary Fig. S4).

In AML with *NPM1* or *RUNX1* mutations the *SF3B1* VAFs exceeded 30% in all cases and were similar to or higher than the VAFs of *NPM1* or *RUNX1* mutations in 11/11 cases (Fig. 2 and Supplementary Figs. S3F and S5A, B). A comparable pattern was seen in the remaining cases with *SF3B1* VAFs higher than 15% (*n* = 29; Fig. 2 and Supplementary Fig. S5C). In the one AML-MRC patient with a low *SF3B1* VAF (6%), other mutations showed higher VAFs (*IDH1*: 42%; *KMT2A*-PTD: 24%; Supplementary Fig. S5C). In total, in 40/41 (98%) *SF3B1*^mut^ cases similar or higher *SF3B1* VAFs were observed compared to other co-mutations or aberrations, indicating that *SF3B1* mutations are rather primary than secondary mutations during leukemogenesis. This is in line with a previous report, showing that *SF3B1* mutations are acquired early in MDS and that splicing mutations are early evolutionary events in myeloid malignancies [14]. In 16/41 (39%) *SF3B1*^mut^ cases molecular follow-up data was available (Fig. 2). In 1/16 patients, an AML patient with mutated *NPM1*, the *SF3B1* mutation (VAF: 40%) remained detectable, despite complete hematologic remission and undetectable *NPM1* mutation (Supplementary Fig. S6B). In 15/16 (94%) cases the *SF3B1* VAFs paralleled the VAFs of co-mutations during the entire disease course, even during relapse (for details see Supplementary Results and Supplementary Fig. S6).

As shown, mutations in *RUNX1* or DTA genes were found among the most frequent co-mutations of *SF3B1*^mut^ AML samples, concordant with previously published studies [5, 14]. Notably, DTA genes are frequently mutated in MDS [6] and the most common mutations in clonal hematopoiesis of indeterminate potential (CHIP) [11]. In our study, a prior history of MDS or MDS/MPN has been documented in some *SF3B1*^mut^ AML patients (8/41). However, it might be unidentified in others. Alternatively, *SF3B1*^mut^ CHIP or CCUS (clonal cytopenia with undetermined significance) may represent relevant precursor lesions of *SF3B1*^mut^ AML, in line with Venable et al., showing that *SF3B1*^mut^ cases comprise the full pathologic spectrum of myeloid disorders from CCUS to AML [14]. In *SF3B1*^mut^ s-AML patients, we frequently detected *MECOM*-r and *RUNX1* mutations, both known AML driver genes [5]. These two genetic abnormalities were also frequently found within the remaining *SF3B1*^mut^ AML patients, where no MDS or MDS/MPN history had been reported. In this line, we previously showed that *SF3B1*^mut^ MDS patients harboring *RUNX1* mutations frequently progressed to AML and that *RUNX1* mutations and *MECOM*-r were gained during AML transformation [15]. Thus, our data suggests an MDS/CCUS pre-phase in *SF3B1*^mut^ AML without antecedent clinical documentation and further supports the guidelines of the WHO 2022 showing that *SF3B1*^mut^ AML is diagnosed as AML-MR without knowing the patient’s clinical history.

In summary, *SF3B1* mutations are found in a small fraction of AML patients, are enriched in poor risk AML subtypes and are strongly associated with *MECOM* rearrangements and myelodysplasia-related changes. The persistently high VAF of *SF3B1* mutations in AML patients suggests that *SF3B1* mutations are acquired early in a pre-leukemic clone and may be indicative of an MDS pre-phase.

## Supplementary information

Supplementary Material

## Data Availability

The datasets generated during and/or analyzed during the current study are available from the corresponding author on reasonable request.
