# Supplementary material for: SF3B1 mutations in AML are strongly associated with MECOM rearrangements and may be indicative of an MDS pre-phase
Source: Leukemia. 2022 Oct 21;36(12):2927–30. doi: 10.1038/s41375-022-01734-7 (PMC9712091; doi:10.1038/s41375-022-01734-7)
Supplement: Supplementary file 1 — Supplementary Material [file 41375_2022_1734_MOESM1_ESM.pdf]

Supplementary Material

Supplementary Methods

Patients cohort and samples

Diagnoses (from peripheral blood and bone marrow) were made based on cytomorphology, cytogenetics, flow cytometry and molecular genetics as previously published [1-3]. The AML cohort comprised 327 (44%) female and 408 (56%) male cases with a median age of 68 years (range: 2-93 years) and a median follow-up of 6 years. Four s-AML patients were already included in a previous study on *SF3B1* mutations analyzing their prior MDS phase [4].

Whole genome sequencing (WGS)

WGS analysis were performed for all patients. For WGS, total genomic DNA was extracted from lysed cell pellet of bone marrow or peripheral blood using the MagNA Pure 96 with DNA and Viral Nucleic Acid Large Volume Kit and Cellular RNA Large Volume Kit (Roche, Basel, Switzerland). Library preparation and sequencing as well as calling and filtering of single nucleotide variants (SNVs), structural variants (SVs) and somatic copy number variations (CNVs) were performed as previously described [5, 6]. Copy neutral loss of heterozygosity (CN-LOH) was assessed using HadoopCNV.

Mutational analysis

In this study, we evaluated mutations in 73 genes associated with myeloid neoplasms for all patients from WGS data only or from combined WGS and targeted NGS panels (*ASXL1, APC, ASXL2, ATM, ATRX, BCOR, BCORL1, BRAF, BRCC3, CALR, CBL, CDH23, CDKN2A, CEBPA, CREBBP, CSF3R, CSNK1A1, CTCF, CUX1, DDX41, DDX54, DHX29, DNMT3A, EP300, ETNK1, ETV6, EZH2, FANCL, FBXW7, FLT3*-TKD*, GATA1, GATA2, GNAS, GNB1, IDH1, IDH2, JAK2, KDM5A, KDM6A, KIT,*

KMT2D, KRAS, MPL, MYC, NF1, NOTCH1, NPM1, NRAS, PHF6, PIGA, PPM1D, PRPF8, PTPN11, RAD21, RB1, RUNX1, SETBP1, SF1, SF3A1, SF3B1, SH2B3, SMC1A, SMC3, SRSF2, STAG2, SUZ12, TET2, TP53, U2AF1, U2AF2, WT1, ZBTB7A, ZRSR2). *KMT2A*-PTD was analyzed with a quantitative PCR assay, *FLT3*-ITD by gene scan, both described methodically previously [7, 8] . Detection limit for *FLT3*-ITD was 5%. From all 735 cases, 325 samples were additionally analyzed by targeted NGS panels during routine diagnostics [9]. WGS data confirmed all mutations detected by targeted NGS and was further consulted for completing the mutational analysis of above mentioned genes. Structural variants/ fusions were analyzed by routine cytogenetics (encompassing chromosome banding analyses and FISH). All cases with 3q26-rearrangements by chromosome banding analyses were analyzed with FISH probes XL t(3;3)GATA2/MECOM DF (MetaSystems, Altlussheim, Germany) and/or XL MECOM (MetaSystems, Altlussheim, Germany) to confirm *MECOM*-rearrangements.

Statistical analysis

All statistical analyses were performed using SPSS version 19.0 (IBM Corporation, Armonk, NY). Analysis for overall survival (OS) was performed according to Kaplan-Meier and compared using the two-sided log rank test. The OS was calculated as the time from diagnosis to death or last follow-up. All results were considered significant at $p < 0.05$.

Supplementary Results

Ring sideroblasts (RS) in *SF3B1*^{mut} AML samples

As RS, which are associated with *SF3B1* mutations in MDS, are not routinely analyzed in AML patients corresponding data was only available in 20/41 *SF3B1*^{mut} AML samples with RS percentages ranging from 0 to 93 (median: 0%; mean: 6%). In *SF3B1*^{mut} AML with maturation (n = 6) in 4 patients the presence of RS was analyzed showing RS in two cases (RS: 4% and 93%).

Recurrent SF3B1 mutations

In the total cohort, 14 different *SF3B1* missense mutations were detected (Suppl. Figure S2). The mean variant allelic frequency (VAF) of the different *SF3B1* mutations ranged from 26% to 51% affecting 9 different amino acids (Suppl. Figure S2A). Within all *SF3B1*^{mut} samples K666 was the most frequently altered amino acid (39%,16/41) while the second most frequently affected one was K700 (34%, 14/41), together accounting for 73% (30/41) of *SF3B1*^{mut} cases (Suppl. Figure S2B). Within each entity the mean VAF of *SF3B1* mutations ranged from 38% to 51% (Table in Suppl. Figure S2B). The VAF of each *SF3B1* mutation did not exceed 54% (Suppl. Figure S2C; range: 6-54%). *SF3B1* VAFs higher than 30% were seen in 85% (35/41), while 12% (5/41) of *SF3B1*^{mut} samples showed VAFs between 15% and 29% (Suppl. Figure S2C, S5C). One AML-MRC case had an *SF3B1* (p.K700E) VAF of 6%. Notably, no CNVs and CN-LOHs overlapping with *SF3B1* were found.

Classification of SF3B1 mutated cases

Based on WHO 2022, 12 cases were assigned as AML-MR, of which 6 were newly classified as AML-MR due to the presence of *SF3B1* mutations (AML with *RUNX1*: n = 4; AML-NOS: n = 2) with 5 cases showing normal karyotypes while one patient with AML with *RUNX1* mutation had an aberrant karyotype (46,XY,del(17)(q11q22)). Following ICC guidelines, *MECOM*-rearranged *SF3B1*^{mut} cases would fall into two

separate sub-groups, i.e. AML with *GATA2::MECOM* and other (specific) *MECOM*-r. Notably, the only *SF3B1*^{mut} sample diagnosed with AML with biallelic *CEBPA* based on WHO 2017 would be defined as AML with MR gene mutations as the patient did not harbor a *CEBPA* mutation in the bZIP domain. One AML-MRC case was classified as AML with mutated *TP53* when considering ICC.

***SF3B1^{mut}* in MDS phase of s-AML patients**

For 5/8 s-AML patients, data from the prior MDS stage was available (Suppl. Figure S4: #1-5). In cases #2 to #5 the *SF3B1* mutation was already present at a high VAF (37%, 44%, 40%, 45%, respectively) at MDS diagnosis, while in case #1 the *SF3B1* mutation was absent in the MDS phase showing a *SF3B1* VAF of 6% at the AML stage (Suppl. Figure S4B-F: #1-5).

Molecular genetics of SF3B1^{mut} patients during disease course

From 15 patients, showing *SF3B1* VAFs parallel to co-mutations, in 4, the VAF of *SF3B1* decreased, similar to accompanying mutations and also to bone marrow blasts, resulting in complete remission (Suppl. Figure S6E, F). In all 5 cases with relapse the *SF3B1* mutation re-occurred at relapse together with other mutations (Suppl. Figure S6C, D). In the remaining cases (n = 7), the *SF3B1* mutation persisted during follow-ups like other aberrations while showing no clinical response in 6/7 cases measured by bone marrow blasts (Suppl. Figure S6A).

Supplementary Table and Figures

Suppl. Table S1. WHO entities and *SF3B1* mutations within the AML cohort

WHO 2017 Diagnosis	Number of samples, n	*SF3B1* mutation = *SF3B1*^{mut}, n (%)
AML with *DEK::NUP214*	15	0 (0)
AML with *KMT2A::MLLT3*	26	0 (0)
AML with *RUNX1::RUNX1T1*	41	0 (0)
AML with *PML::RARA*	48	1 (2)
AML with *CBFB::MYH11*	45	1 (2)
AML with *GATA2::MECOM*	36	10 (28)
AML with biallelic mutation of *CEBPA*	47	1 (2)
AML with mutated *NPM1*	162	5 (4)
AML with mutated *RUNX1**	51	6 (10)
AML with myelodysplasia-related changes (AML-MRC)	158	11 (7)
AML, not otherwise specified (AML-NOS)	106	6 (6)
AML with minimal differentiation	10	0 (0)
AML without maturation	16	0 (0)
AML with maturation	32	6 (19)
Acute myelomonocytic leukemia	33	0 (0)
Acute monoblastic and monocytic leukemia	14	0 (0)
Pure erythroid leukemia	1	0 (0)
**AML total**	**735**	**41 (6)**

* provisional entity

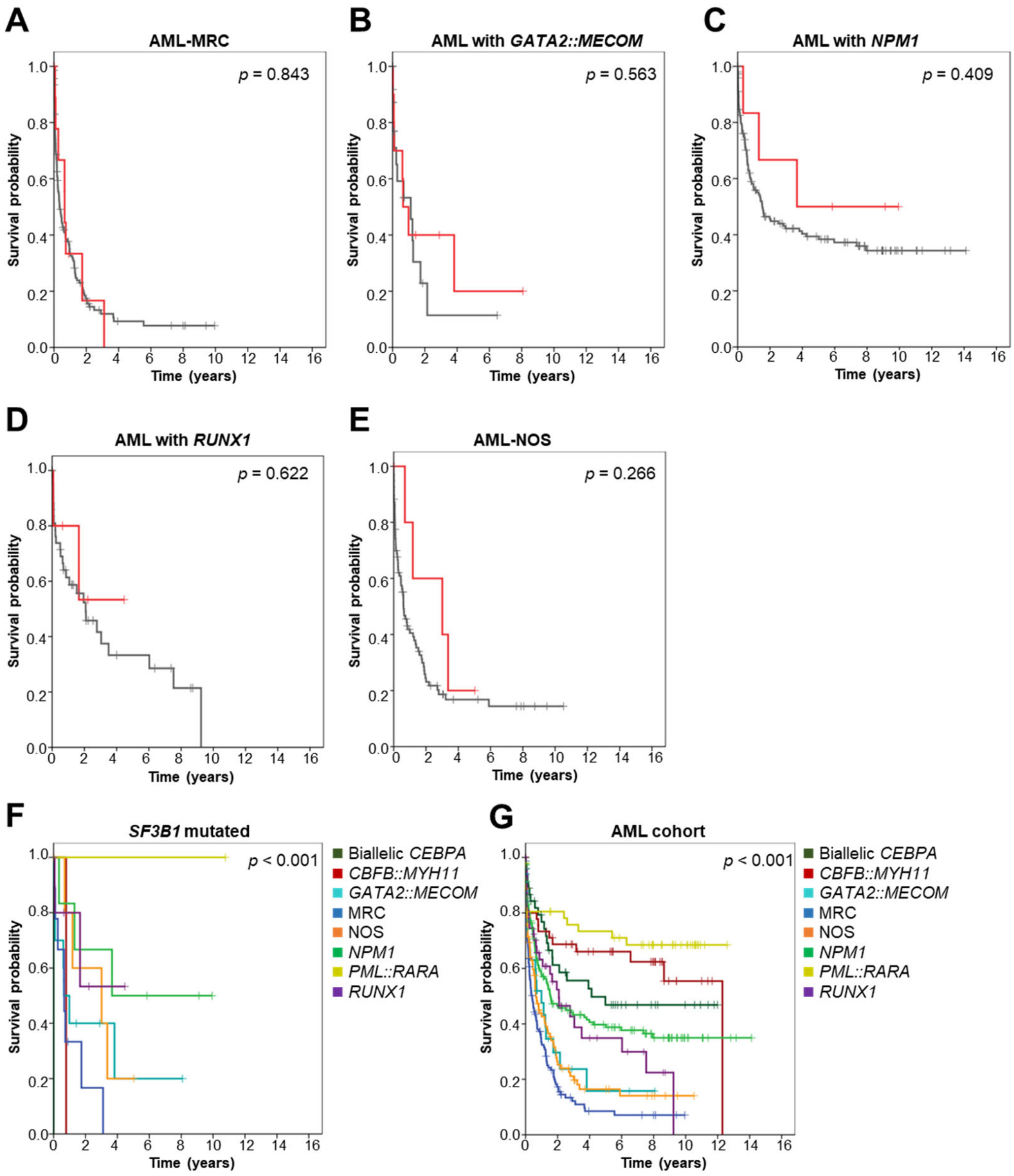

Suppl. Figure S1: Overall survival (OS) of *SF3B1* mutations in AML entities. (A) OS of AML-MRC with mutated (n = 11; red) vs. wild-type (n = 147; grey) *SF3B1*; median OS: 8 vs. 4 months. (B) OS of AML with *GATA2::MECOM* with mutated (n = 10; red) vs. wild-type (n = 26; grey) *SF3B1*; median OS: 8 vs. 13 months. (C) OS of AML with *NPM1* with mutated (n = 6; red) vs. wild-type (n = 156; grey) *SF3B1*; median OS: 44 vs. 18 months. (D) OS of AML with *RUNX1* with mutated (n = 5; red) vs. wild-type (n = 46; grey) *SF3B1*; median OS: not reached vs. 25 months. (E) OS of AML-NOS with mutated (n = 6; red) vs. wild-type (n = 100; grey) *SF3B1*; median OS: 36 vs. 7 months. (F) OS of *SF3B1* mutated AML according to WHO 2017 entities. (G) For

comparison to figure (F) OS of all AML cases assigned to one of the 8 WHO 2017 entities in which *SF3B1* mutated cases were observed is depicted.

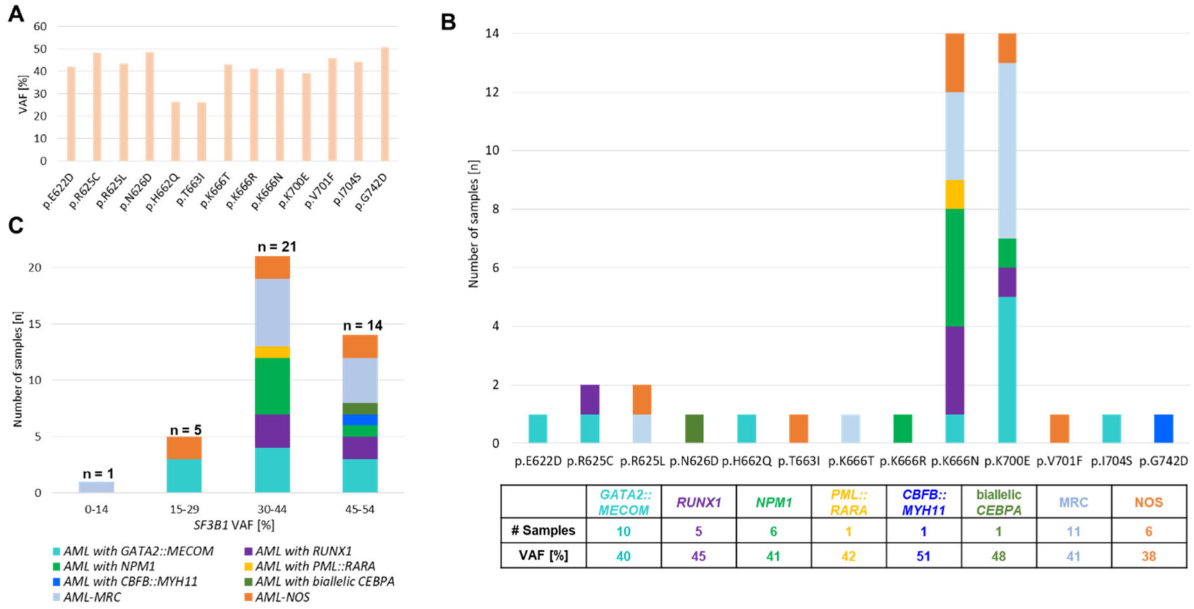

Suppl. Figure S2: Variety of *SF3B1* mutations in AML. (A) Average variant allelic frequency (VAF) of different *SF3B1* mutations (n = 41). (B) Frequency of *SF3B1* mutations within all *SF3B1* mutated samples (n = 41). (C) *SF3B1* VAFs with respect to the different entities.

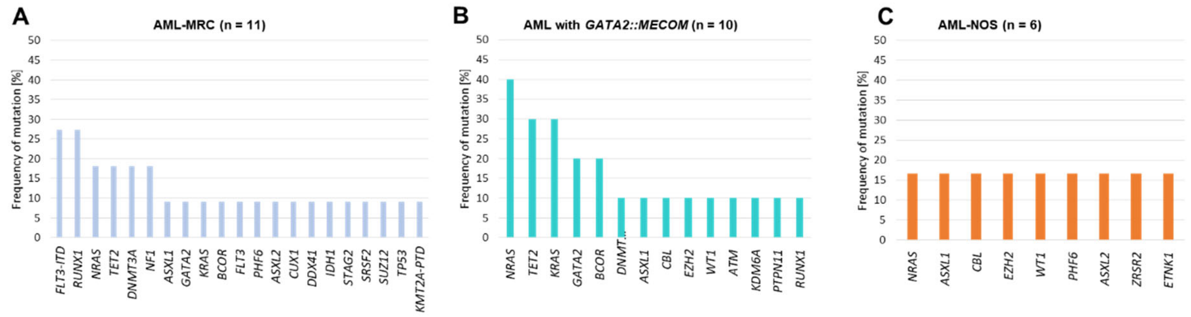

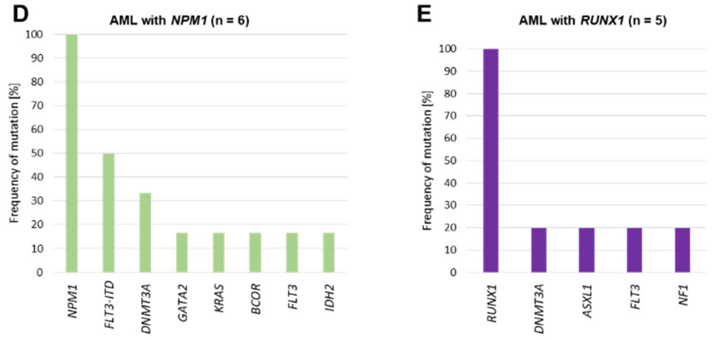

F

	VAF [%]		
	SF3B1	*NPM1*	*RUNX1*
AML with *NPM1*	**42**	34	
AML with *RUNX1*	**45**		28

Suppl. Figure S3: Additional gene mutations in *SF3B1* mutated patients. Frequency of additional gene mutations within AML-MRC (A), AML with *GATA2::MECOM* (B), AML-NOS (C), AML with NPM1 (D) and AML with *RUNX1* (E). (F) Mean variant allelic frequency (VAF) of *SF3B1* and *NPM1* or *RUNX1* in corresponding entity.

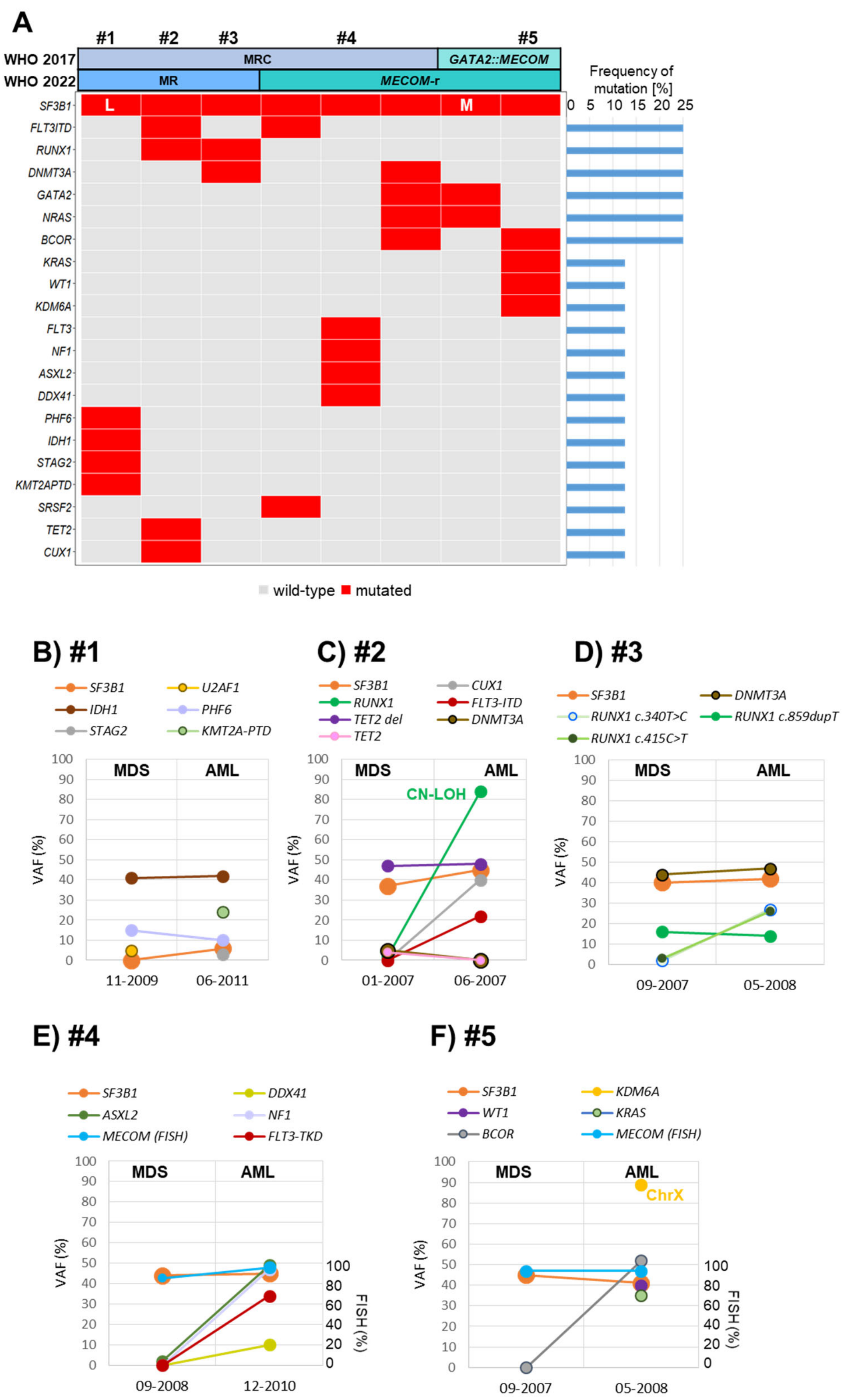

Suppl. Figure S4: Molecular characterization of *SF3B1*^{mut} AML patients with a prior history of MDS or MDS/MPN. (A) Illustration of all 8 samples, each column represents one patient. Genes (grey: wild-type; red: mutated) as well as the WHO

entities are given for each patient. MR(C): myelodysplasia-related (changes); *MECOM*-r: *MECOM* rearrangement. Patient with available data from prior MDS stage are marked with #. L: low VAF (0-14%); M: medium VAF (15-29%); Remaining cases showed *SF3B1* VAFs ≥ 30%. (B-F) Genetic evolution of patients #1-5 from panel A with molecular data from prior MDS phase. VAF: variant allelic frequency; FISH: fluorescence in situ hybridization; CN-LOH: copy neutral loss of heterozygosity; ChrX: X-linked gene/ male patient.

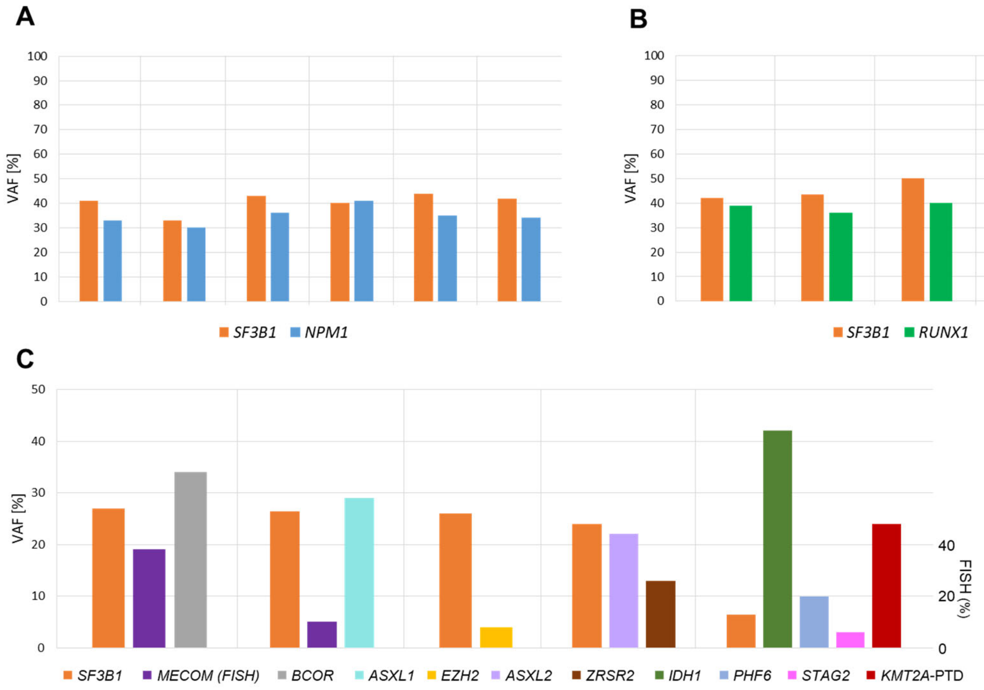

Suppl. Figure S5: Molecular genetics at AML diagnosis. (A) VAFs of *SF3B1* compared to *NPM1* in AML with mutated *NPM1* (n = 6). (B) VAFs of *SF3B1* compared to *RUNX1* in AML with mutated *RUNX1* (n = 5). (C) VAFs of *SF3B1* compared to co-mutations in patients with a *SF3B1* VAF < 30% (n = 5). VAF: variant allelic frequency; FISH: fluorescence in situ hybridization; CN-LOH: copy neutral loss of heterozygosity.

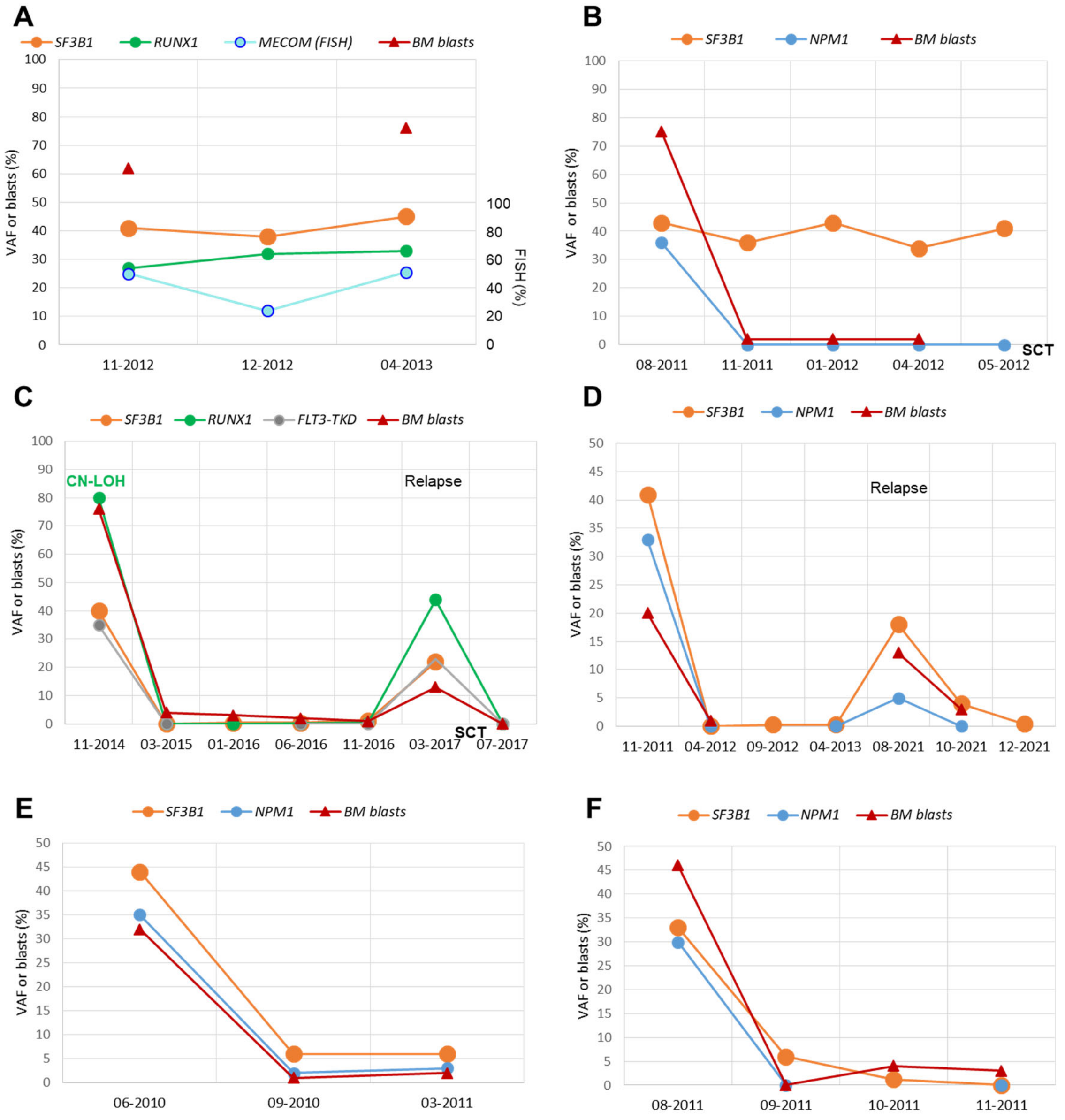

Suppl. Figure S6: Molecular genetics during follow up. Representative examples for genetic evolutions of a patient with AML with *GATA2::MECOM* (A), AML with mutated *NPM1* (B, D, E, F) and AML with mutated *RUNX1* (C) during disease courses. The *SF3B1* mutation behaved like other mutations in A, C, D, E and F. In B *SF3B1* persisted while other mutations decreased. VAF: variant allelic frequency; BM: bone marrow; FISH: fluorescence in situ hybridization; CN-LOH: copy neutral loss of heterozygosity; SCT: stem cell transplantation.

References

1. Schoch C, Schnittger S, Bursch S, Gerstner D, Hochhaus A, Berger U, et al. Comparison of chromosome banding analysis, interphase- and hypermetaphase-FISH, qualitative and quantitative PCR for diagnosis and for follow-up in chronic myeloid leukemia: a study on 350 cases. Leukemia. 2002;16(1):53-9.
2. Haferlach T, Kern W, Schoch C, Hiddemann W, Sauerland MC. Morphologic dysplasia in acute myeloid leukemia: importance of granulocytic dysplasia. J Clin Oncol. 2003;21(15):3004-5.
3. Kern W, Voskova D, Schoch C, Hiddemann W, Schnittger S, Haferlach T. Determination of relapse risk based on assessment of minimal residual disease during complete remission by multiparameter flow cytometry in unselected patients with acute myeloid leukemia. Blood. 2004;104(10):3078-85.
4. Huber S, Hutter S, Meggendorfer M, Hoermann G, Walter W, Baer C, et al. The Role of SF3B1 Mutations in Myelodysplastic Syndromes. EHA Library. 2022;357597:P735.
5. Höllein A, Twardziok SO, Walter W, Hutter S, Baer C, Hernandez-Sanchez JM, et al. The combination of WGS and RNA-Seq is superior to conventional diagnostic tests in multiple myeloma: Ready for prime time? Cancer Genet. 2020;242:15-24.
6. Stengel A, Baer C, Walter W, Meggendorfer M, Kern W, Haferlach T, et al. Mutational patterns and their correlation to CHIP-related mutations and age in hematological malignancies. Blood Adv. 2021;5(21):4426-34.
7. Schnittger S, Kinkelin U, Schoch C, Heinecke A, Haase D, Haferlach T, et al. Screening for MLL tandem duplication in 387 unselected patients with AML identify a prognostically unfavorable subset of AML. Leukemia. 2000;14(5):796-804.

8. Schnittger S, Schoch C, Kern W. FLT3 length mutations in AML: correlation to cytogenetics, FAB-subtype and prognosis in 652 patients.(Abst). Blood. 2000;96:826a.

9. Meggendorfer M, Haferlach C, Kern W, Haferlach T. Molecular analysis of myelodysplastic syndrome with isolated deletion of the long arm of chromosome 5 reveals a specific spectrum of molecular mutations with prognostic impact: a study on 123 patients and 27 genes. Haematologica. 2017;102(9):1502-10.
